# The complete chloroplast genome sequence of *Malus toringoides* (Rosaceae)

**DOI:** 10.1080/23802359.2020.1780977

**Published:** 2020-07-14

**Authors:** Yanan Li, Yanlei Liu, Ping Wu, Sheng Zhou, Ling Wang, Shiliang Zhou

**Affiliations:** a College of Landscape Architecture, Northeast Forestry University, Harbin, China; b State Key Laboratory of Systematic and Evolutionary Botany, Institute of Botany, Chinese Academy of Sciences, Beijing, China; c College of Life Sciences, University of Chinese Academy of Sciences, Beijing, China

**Keywords:** *Malus toringoides*, Amygdaloideae, Rosaceae, complete chloroplast genome, phylogenetic

## Abstract

*Malus toringoides* belongs to the subfamily Amygdaloideae of Rosaceae, which is an endemic species in China. It has significant ornamental, economic, and ecological value. Herein, we assembled the complete chloroplast genome of *Malus toringoides* using the next-generation sequencing technology. The total length of the complete chloroplast genome was 160,093 base pair (bp), consisting of one large single-copy (LSC) region with a sequence length of 88,177 bp, one small single-copy (SSC) region which sequence length is 19,194 bp, and a pair of inverted repeat regions (IRs, 26361 bp). Besides, the complete chloroplast genome contained 128 genes, namely 83 protein-coding genes (PCGs), 37 tRNA genes (tRNA), and 8 rRNAgenes (rRNA), the GC content was 36.6%. The phylogenetic relationship among species in genus *Malus* is closely related, especially the phylogenetic relationship among *Malus angustifolia, Malus prattii, Malus micromalus, Malus prunifolia, Malus baccata, Malus hupehensis* and *Malus toringoides*. Furthermore, phylogenetic relationship of *Malus toringoides* in *Malus* was closely related to *Malus hupehensis*. Our study affords important genetic information for further researches on the related species.

## Introduction

*Malus*, a genus, belongs to the Rosaceae family, which includes approximately 35 species all over the world. The majority of the species grow in the temperate regions of the Northern Hemisphere. There are 23 species in China and most of them possess important ornamental, economic, and ecological value (Tang et al. 2015). Among them, *Malus toringoides* is one of the seven endemic species in China (Qian 2005; Wu and Hong 2009). Apples are suitable for hillside terraces, plain wilderness and loess hills, with an elevation of 50–2500 meters (Wu and Hong 2009). Due to these unique merits in physiological features, the species in this genus are ideal garden greening and important honey source for insects. Contributing to the widespread hybridization between species within genus *Malus*, the species relationship among this genus is confused. Therefore, we hope to explore the phylogenetic relationship in *Malus* based on the sequences of chloroplast genome.

Total genomic DNA was extracted from the silica gel dried and clean leaves of *Malus toringoides* sampled from Wuhan Botanical Garden (China; N30°31′21.62″, E114°25′34.99″). Meanwhile, the voucher specimen (POC529082) was deposited in the herbarium, Institute of Botany, Chinese Academy of Sciences (PE). Total DNA was extracted via modified cetyltrimethyl ammonium bromide (mCTAB) method (Li et al. 2013). An NGS library was constructed and sequenced at Beijing Novogene Bioinformatics Technology Co., Ltd (Beijing) on Illumina HiSeq2500 platform using pair-end 150 bp (PE150) strategy. Twenty millions sequence reads were obtained and chloroplast genome reads were sorted out and the genome was assembled *de novo* using SPAdes 3.9 (Bankevich et al. 2012). Finally, the assembled chloroplast genome was then annotated on Plann with *Malus angustifolia* (No.NC045410) as reference and corrected with Sequin (Huang and Cronk 2015). And then, the annotated chloroplast genome sequences were submitted to GenBank (accession number: MT483999).

The complete chloroplast genome of *Malus toringoides* is 160,093 bp in length. It includes two copies of inverted repeats (IRs, 26361 bp), a large single-copy (LSC, 88177 bp), and a small single-copy (SSC, 19194 bp) regions. A total of 128 genes were annotated, containing 83 protein-coding genes (PCGs), 37 transfer RNA genes (tRNA), and 8 ribosomal RNA genes (rRNA). Overall GC content was 36.6% and those in LSC, SSC, and IR regions were 34.2%, 30.4%, and 42.7%, respectively.

**Figure 1.**
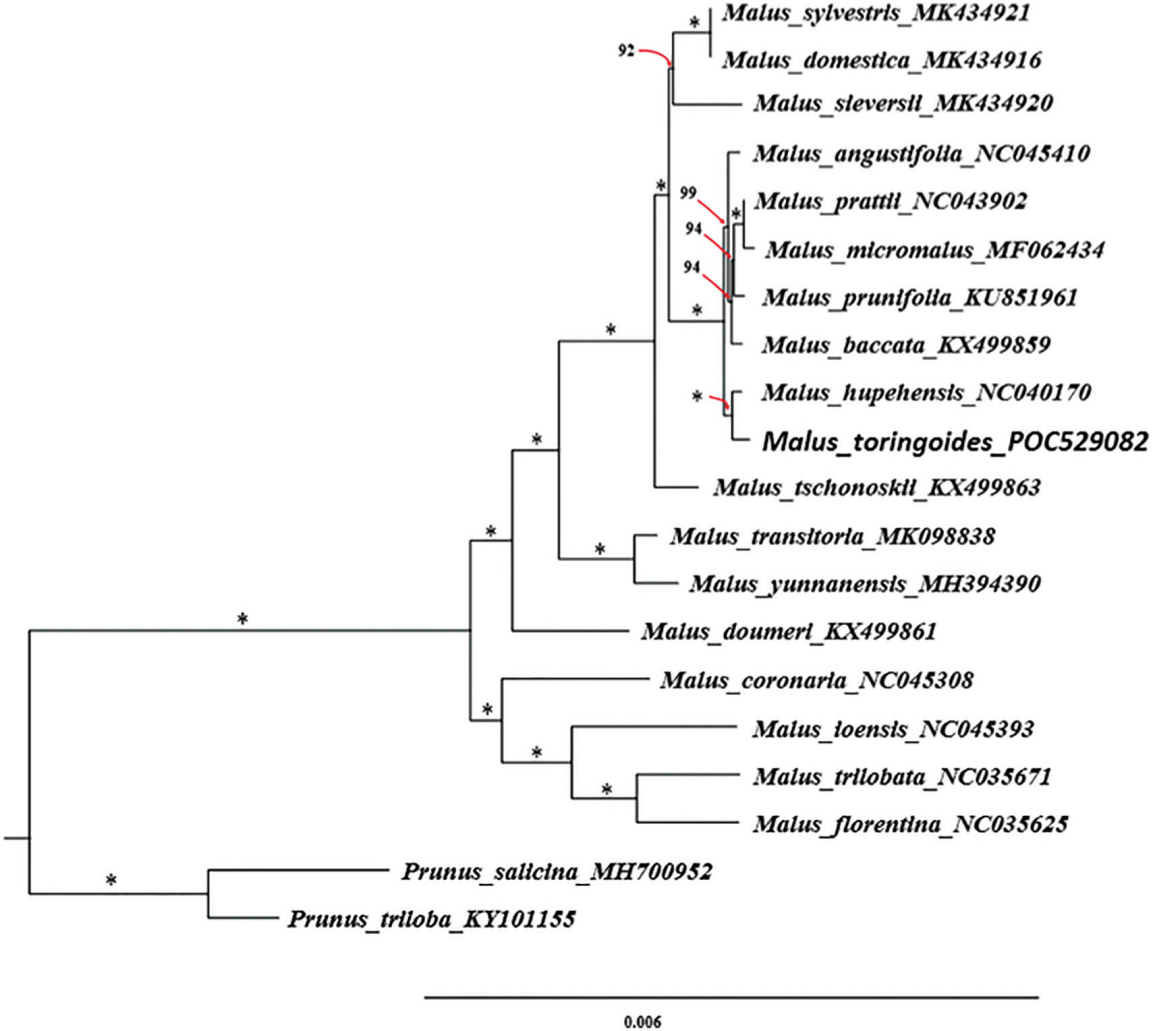
Phylogenetic tree based on plastid genomes using the ML method. Ultrafast bootstrap (UFBoot) values are shown above the nodes, with 1000 bootstrap replicates. *Represents that this result is 100% supported.

A phylogenetic analysis was carried out using whole chloroplast genome sequences of *Malus toringoides* and other 17 chloroplast genome sequences of species in *Malus* which could be downloaded from the NCBI database. Meanwhile, two species from the Prunus were used as outgroups. Multiple sequences alignment was executed by MAFFT (Katoh and Standley 2013). ModelFinder was used for model selection according to the Bayesian information criterion (BIC) (Kalyaanamoorthy et al. 2017). A maximum likelihood (ML) tree with 1000 bootstrap replicates were inferred by IQ-TREE (Nguyen et al. 2015). The phylogenetic result indicates that the phylogenetic relationship among species in genus *Malus* is closely related, especially among species *Malus angustifolia, Malus prattii, Malus micromalus, Malus prunifolia, Malus baccata, Malus hupehensis* and *Malus toringoides*. The relationship within genus *Malus* can be well distinguished using whole chloroplast genome data. *Malus toringoides* is most closely related to *Malus hupehensis* (100% ultrafast bootstrap support, Figure 1) (Hoang et al. 2018). This result would be beneficial to potential studies on phylogenetics of the genus and related group in Rosaceae.

## Data Availability

The data that support the findings of this study are openly available in NCBI GenBank database at (https://www.ncbi.nlm.nih.gov) with the accession number is MT483999, which permits unrestricted use, distribution, and reproduction in any medium, provided the original work is properly cited.
